# Anapole state revealed by cloaking metallic cylinders with split ring resonators

**DOI:** 10.1038/s41598-023-43917-x

**Published:** 2023-10-05

**Authors:** Valiyaveettil Pushpakaran Sarin, Giuseppe Labate, Puthiyapurayil Viswanathan Vinesh, Manoj Mani, Pezholil Mohanan, Vasudevan Kesavath

**Affiliations:** 1 Department of Electronics, Government Arts and Science College Tanur, Malappuram, Kerala India; 2 Wave-Up, Innovation in Electromagnetics, Siena, Italy; 3 Centre for Research in Electromagnetics and Antennas, Cochin University of Science and Technology, Cochin, Kerala 682022 India

**Keywords:** Electrical and electronic engineering, Applied physics, Condensed-matter physics, Condensed-matter physics

## Abstract

This paper proposes the experimental demonstration of an anapole-based cylindrical electromagnetic cloaking scheme. An anapole state is excited by arranging around a cylindrical metallic target vertical split-ring resonators, forming an equivalent surface admittance boundary condition able to suppress the scattering. Using Mie formalism and multipole scattering theory, we identify the actual reason behind the cloaking operation, characterizing the anapole condition by the scattering contributions from toroidal and electric dipole moments. Numerical results are verified using full-wave simulation softwares and subsequently validated with back-scattering measurements inside an anechoic chamber.

## Introduction

The first theoretical paper on loading dielectric cylinders with a metallic core appeared in 1967^1^, representing an early scattering cancellation-based method for what has been referred later as invisibility *cloaking*^2–10^. This term refers to the significant reduction of the scattering cross section (SCS) from reference metallic or dielectric targets and its experimental realization has attracted much research interest in terms of the electromagnetic mechanism and materials behind. The problem addressed in this paper is the reduction of the SCS of a metallic cylinder with a particular architecture: the split ring resonators (SRR). Initial works on SCS reduction were based on the fact that, when the electric and magnetic dipoles induced on a subwavelength dielectric sphere oscillate out-of-phase, the SCS of the composite will be significantly reduced^11^. This conclusion is known as Kerker’s paradox, however, the problem with this scheme is the unavailability of natural magnetic materials used to create magnetic dipole moments at microwave frequencies. It has been showed that the SRRs made of concentric metallic split rings could achieve artificial magnetism at microwave frequencies^12^. This mechanism has been treated as a paradigm shift in electromagnetics because it has been possible to control the amplitude and phase of electric and magnetic moments individually to achieve unprecedented control over electromagnetic scattering. The actual mechanism is introducing circulating currents that are uncompensated, due to the splits in the two double rings, in order to form a non-zero magnetic moment due to symmetry breaking. Applications of such metamaterials include improving the gain of antennas^13^. The first practical demonstration has been proposed in 2006 for cloaking a copper cylinder using single SRR metamaterials: the combination of the target and the metamaterial creates an electromagnetic environment similar to free space^14^, named coordinate transformation or transformation optics^2,3^.

**Figure 1:**
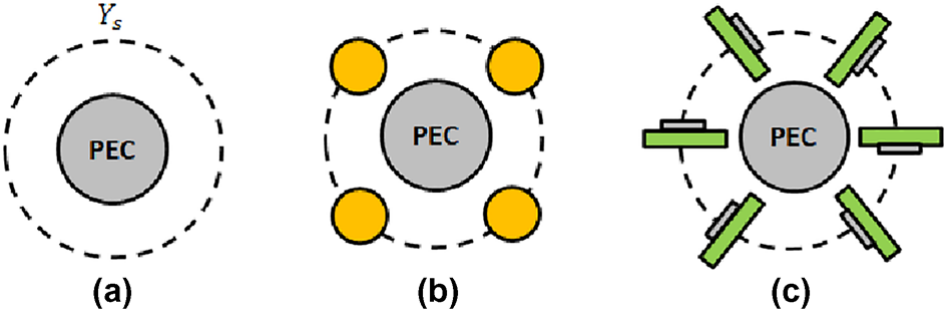
Sketch of metallic cloaking with surface admittance (**a**) and related surface admittance boundary conditions realized with four dielectric pillars^20^ (**b**) and proposed cloak with printed SRR (**c**).

The year before, in 2005, scattering cancellation-based cloaking has been proposed implementing the cloaking mechanism using plasmonic covers over dielectric objects^4^. The dielectric target’s scattered field is cancelled in plasmonic cloaking due to the anti-phase scattering from the negative or less-than-one permittivity of the outer layer. In 2009, a mantle cloaking technique has been reported to cloak both dielectric and metallic targets, and its basic idea relies on tuning the surface reactance of a frequency selective surface (FSS) layer to cancel far-field scattering^5^. Such surface admittance boundary condition has been applied recently to dielectric objects^15^ and metallic scatterers^16^ with closed-form solutions and it is here reported for the case of metallic scattering, in order to compare this equivalent surface admittance boundary condition with its practical realization in terms of properly arranged dielectric and metallic materials, as reported in Fig. 1. Recently, a super-dispersion-based broadband electromagnetic cloak operating at terahertz frequencies has got considerable attention^17^. Other approaches in the form of covering the metallic target with dielectric materials^18^ and corrugating the metallic target^19^ are also used for efficient cloaking in the microwave regime in which the scattering from the fundamental electric dipole moments is suppressed using additional resonances. In the recent literature^20^ four dielectric pillars have been arranged around a metallic cylinder to cloak it from an incoming external plane wave. The phenomenon of invisibility cloaking has been explained there as the excitation of a higher-order multipole, known as the toroidal moment. In particular, toroidal dipoles are created due to the poloidal currents flowing on the meridians of a toroid^21,22^, and their scattering behavior is similar to that of an electric dipole^23^. These currents were created by coupling the magnetic moment of the four dielectric pillars in a loop, however, in this paper, these currents flowing on the meridians of a toroid have been implemented directly with split-ring resonators, since the excitation of toroidal moments adds an additional degree of freedom to control electromagnetic scattering. Since the scattering characteristics of the electric dipole and the toroidal dipole are similar, an out-of-phase excitation of these moments results in invisibility cloaking: this proper combination of toroidal moment ($\vec {T}$) and electric dipole moment ($\vec {P}$) is termed as an *anapole*.

Recently, a wide variety of works has been focused on the anapole excitation in metamaterials^24–26^ and for the first time, anapole has been related to cloaking^20^ and to non-radiating currents^27^. There is a close relationship between the Devaney–Wolf theorems regarding non-radiating currents and the anapole mode^27^, since an equivalent current distribution $\vec {J}_{eq}$ can be written in the form

1 $$\begin{aligned} \vec {J}_{eq}=\nabla \times \nabla \times \vec {T} -j k_0\vec {P}. \end{aligned}$$

When Eq. (1) is subject to the condition $\vec {P}=-jk_0\vec {T}$, a Helmholtz-type solution is formed and $k_0$ is the wavenumber at the frequency where anapole may happen.

The novelty of the present work is to link the cloaking mechanism of SRR with the excitation of the anapole mode. Results confirm the findings in the literature^20^ and enlarge the architectures that can be used to excite the anapole mode after a proper multipole expansion is performed. SRRs, at the basis of the transformation optics technique^2,3^, are here used for the first time to excite non-radiating anapole modes if arranged around a metallic cylinder. Here instead of cloaking with four dielectric pillars^20^, a relation between anapole state and surface admittance mantle cloaks^5^ is first suggested and a SRR architecture for the cloak, as in coordinate transformation technique^14^, is proposed.

The paper is organized as follows: in “Surface admittance cloaking with Mie theory” section, Mie theory is used to compute the surface admittance needed to cancel the cylindrical harmonic wave *m*; in “Geometry and full-wave simulations” section, full-wave simulations have been reported for the case of cloaking a metallic cylinder via the proper surface admittance and its realization with SRR, as a function of the vertical SRR layers; in “Experimental prototype” section, experimental setup is assessed and used to verify in anechoic chamber the cloaking phenomenon; in “Discussions with Multipole theory and parametric analysis” section, multipole theory is employed to extract electric dipole, toroidal and magnetic dipole moments to compute the anapole condition. Conclusions and further extension of this work are indicated in “Conclusions and future work” section.

## Surface admittance cloaking with Mie theory

Let us consider a metallic cylinder of radius *a*, with axis aligned to ${\hat{z}}$, loaded by an impedance sheet detached at its surface at a boundary $r=b$. The overall scattering, according to Mie Theory under TM$_ z$ illumination, in a cylindrical coordinate system, can be expanded in cylindrical harmonics as^16,28^

2 $$\begin{aligned} f(r,\phi )= \sum \limits _{m=-\infty }^{m=+\infty }j^{m} f_m(r)e^{-j m\phi }, \end{aligned}$$

where the function $f(r,\phi )$ can represent the electric or magnetic field. In particular the radial coefficients $f_m(r)$ for the electric fields are

3 $$\begin{aligned} \text{E}^z_m = {\left\{ \begin{array}{ll} \ 0 &{} r< a \\ \\ \ [b_m H_m^{(1)}(k_0 r)+d_m H_m^{(2)}(k_0 r) ]\ {} &{} a<r<b \\ \\ \ [J_m(k_0 r)+c_m H_m^{(2)}(k_0 r) ]\ {} &{} r>b \end{array}\right. }, \end{aligned}$$

where $k_0$ is the wavenumber in the entire free-space region. The magnetic field can be directly derived from Maxwell’s equations as $H=j\nabla \times E/\omega \mu _0$ and here reported only the tangential component ${\hat{\phi }}$ at the boundary, being $H^\phi _m$ the radial coefficients

4 $$\begin{aligned} -j\text{H}^\phi _m = {\left\{ \begin{array}{ll} \ Y_0[b_m H_m^{(2)'}(k_0 r)+d_m H_m^{(2)'}(k_0 r) ]\ \ {} &{} a<r <b \\ \\ \ Y_0[J'_m(k_0 r)+c_m H_m^{(2)'}(k_0 r) ]\ \ {} &{} r>b \end{array}\right. }, \end{aligned}$$

where $Y_0$ is equal to $k_0/\omega \mu _0$. Throughout the paper, the time convention $e^{+j\omega t}$ is tacitly assumed. The three unknown expansion coefficients $b_m$ , $c_m$ and $d_m$ represent the scattering coefficients related to the cylindrical harmonics *m* (in 2D $m=0$ is monopole radiation, $m=1$ is dipole, $m=2$ is quadrupole and so on). At the boundary $r=a$ the tangential electric field is continuous due to the absence of magnetic surface sources and the two conditions to compute the unknowns are

5 $$\begin{aligned}&0 = b_m H_m^{(1)}(k_0 a)+d_m H_m^{(2)}(k_0 a), \end{aligned}$$

6 $$\begin{aligned}&J_m(k_0 b)+c_m H_m^{(2)}(k_0 b) =b_m H_m^{(1)}(k_0 b)+d_m H_m^{(2)}(k_0 b), \end{aligned}$$

being a relation between $b_m$ and $d_m$, where $H_m^{(1)}(\cdot )$ and $H_m^{(2)}(\cdot )$ are the *m*-th order Hankel functions of the first (ingoing) and second kind (outgoing), respectively. It is useful to define the ratio $b_m/d_m$ in the sense of a cylindrical reflection coefficient as7where  is defined as $k_0 a$ as in^15^. As reported in the literature^27^, we enforce a proper surface electric current $J_s$ to be equal to the jump of the magnetic fields at $r=b$ as8

The function $g(\cdot )$ depends on the internal and external scattering coefficient, the geometry in terms of wavelength  as

9 $$\begin{aligned} g(\cdot )=J'_m (k_0 b)+c_m H_m^{(2)'}(k_0 b)-b_m H_m^{(1)'}(k_0 b)-d_m H_m^{(2)'}(k_0 b), \end{aligned}$$

representing the jump of the magnetic fields at the boundary. The surface currents are related to the tangential electric field via a scalar surface admittance being

10 $$\begin{aligned} J_s (\phi )= Y_s E_z(r=b,\phi )= Y_s \sum \limits _{m=-\infty }^{m=+\infty }j^{m} \ [J_m(k_0 b)+c_m H_m^{(2)}(k_0 b)]\ e^{-j m\phi }. \end{aligned}$$

Reformulating the three unknowns $Y_s$, $b_m$ and $\gamma _m$ and recalling that for cloaking purpose $|c_m|=0$, the surface admittance can be rewritten as follows, exploiting (5) and (6)11where ${\widetilde{Y}}_s$ is the normalized admittance with respect to $Y_0$. The function $\Delta _m$ is equal to12

**Figure 2:**
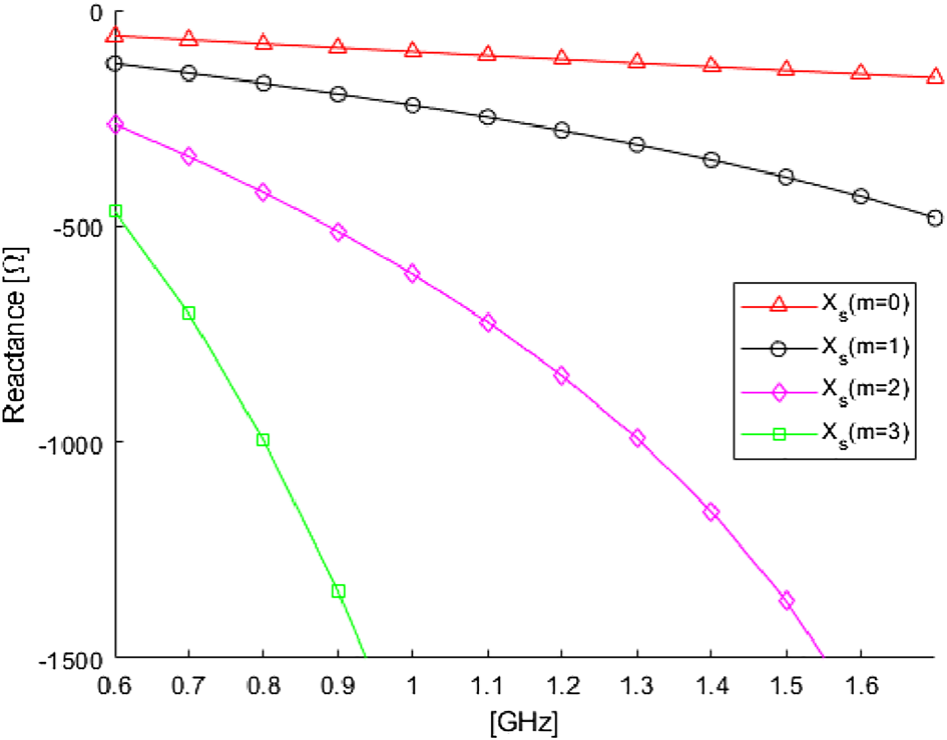
Behaviour of the surface reactance dispersion for each separate harmonic mode to suppress.

Note that for the single harmonic case, the complex value admittance gets simplified and reduces to ${\widetilde{Y}}_s=-j\Delta _m$, which becomes purely imaginary if $\Delta _m$ is real. The second term in Eq. (12) represents in transmission line theory a short circuit (metallic cylinder) as seen from a free-space piece of transmission line from $r=a$ to $r=b$, whereas the first term in Eq. (12) is the purely imaginary admittance of a piece of free-space at $r=b$. In Fig. 2, it is reported the value of the imaginary part of the surface impedance in frequency as a function of the harmonic index to suppress. As expected in subwavelength condition, the metallic cylinder behaves as an inductor, thus its scattering is canceled by a capacitive load^29^. The dispersion behaviour is plotted as a function of the harmonic index to suppress: the higher the harmonics, the higher the absolute value of the reactance needed to cancel the scattering according to Eq. (12). Having such closed-form solution for the surface impedance is a powerful tool to control the scattering features of metallic objects. In the next section, we will enforce a surface admittance value computed using Eq. (12) to an equivalent boundary $r=b$ and compared its electromagnetic fields with respect to the SRR cloak.

### Geometry and full-wave simulations

**Figure 3:**
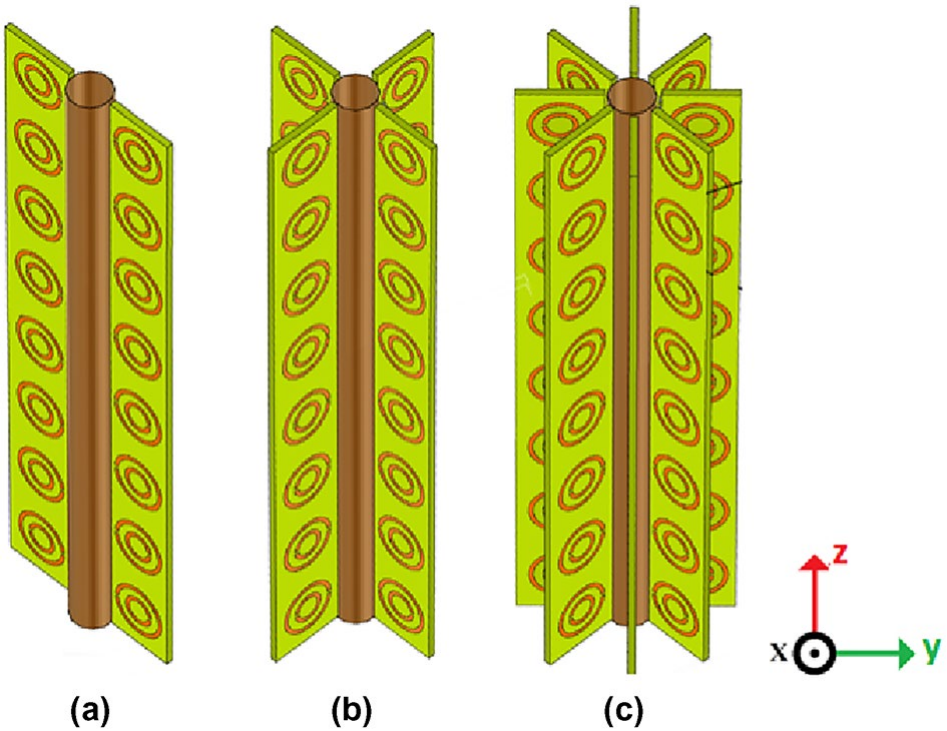
Geometry of the cloaking scheme (**a**) two-layer cloak (case 1), (**b**) four-layer cloak (case 2), and (**c**) eight-layer cloak (case 3).

**Figure 4:**
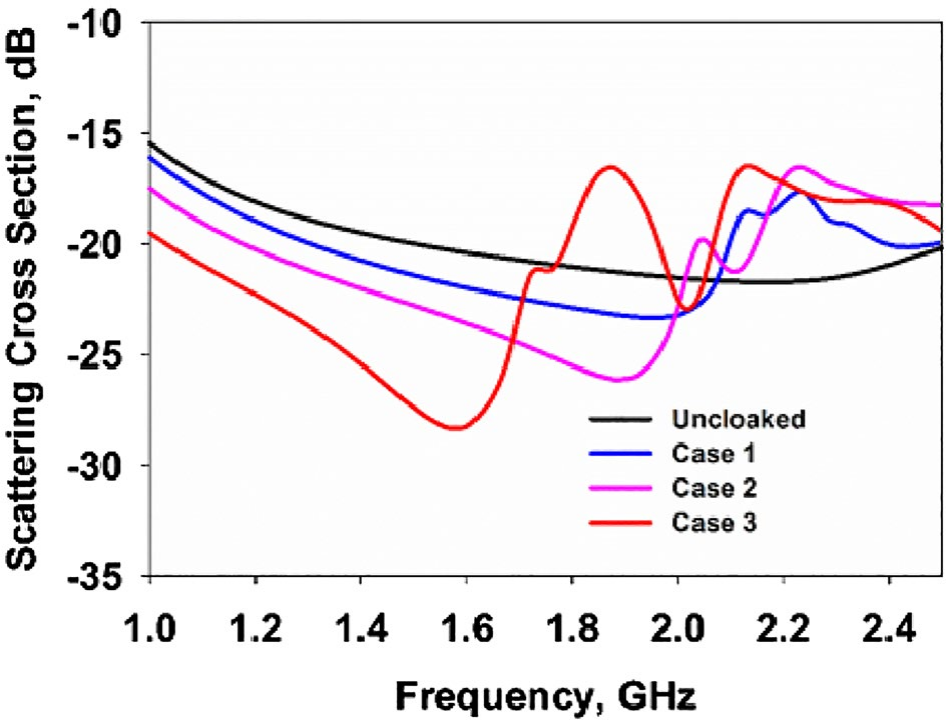
Scattering cross sections of the three prototypes.

**Figure 5:**
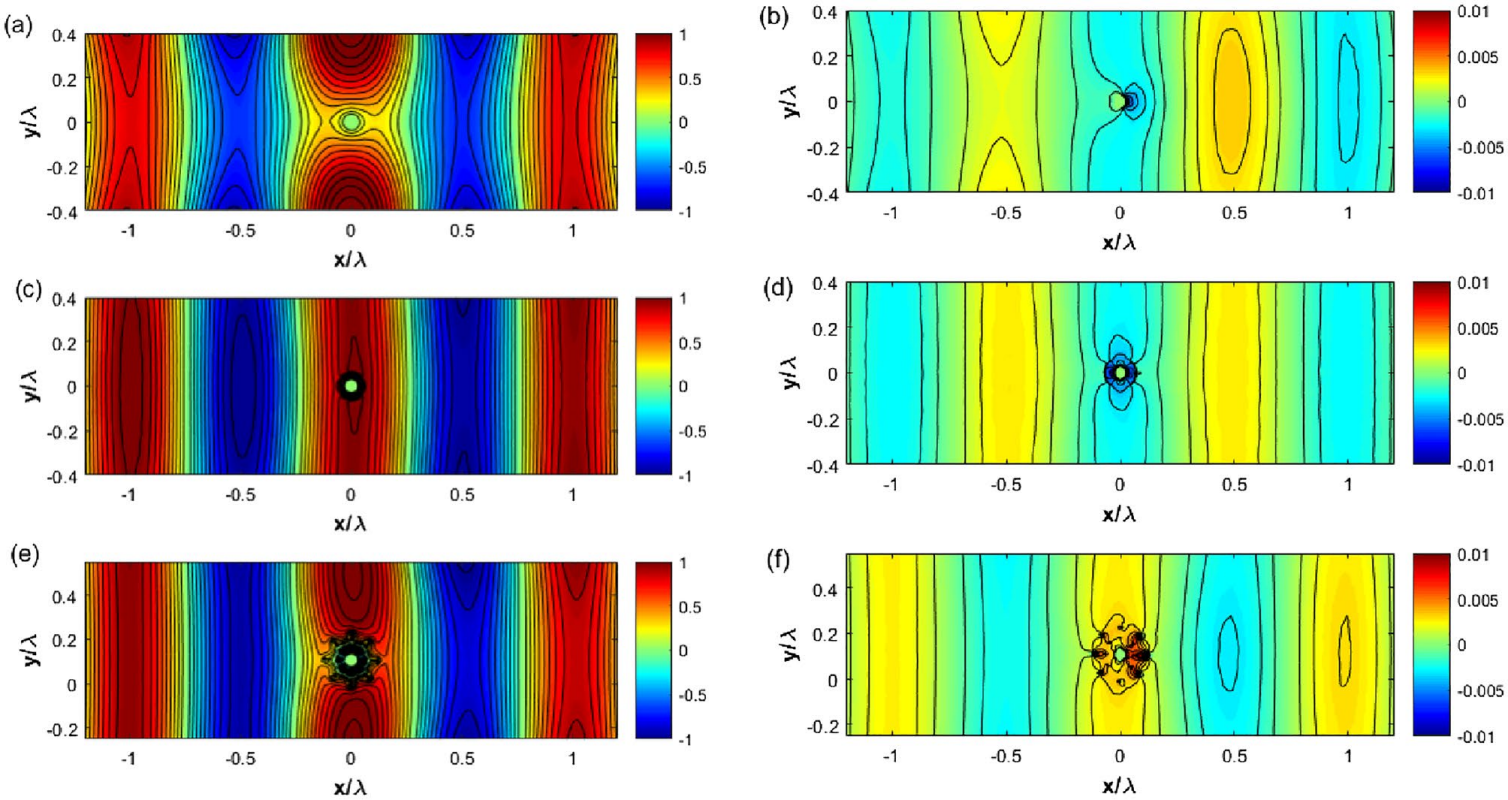
Results of numerical simulation for the real part of total electric (left column) and magnetic field (right column) at 1.6 GHz for the uncloaked target (**a,b**), cloaked with constant surface impedance at $b=13$ mm with $Z_s = - j148.8 \Omega$ (**c,d**) and cloaked with SRR cloak (**e,f**).

The geometry under consideration is a metallic cylinder of radius $a=5$ mm covered by vertical layers of a printed SRR cloak with their centers passing from a circle of radius $b=13$ mm, as shown in Fig. 5. The performances of the SRR cloaks are evaluated considering the SCS, here computed for the three structures, illustrated in Fig. 4, defined in terms of Radar Cross Section (RCS) for 2D cylinders as

13 $$\begin{aligned} RCS(\phi )=\lim _{R\rightarrow \infty } 2\pi R\dfrac{|E_s(R,\phi )|^2}{|E_i(R,\phi )|^2}, \end{aligned}$$

and thus directly proportional to Mie cattering coefficient $|c_m|^2$. The SCS is the average value over $2\pi$ of the RCS. As reported in Figs. 3 and 4, the considered cases 1, 2 and 3 are here indicated as having number of vertical SRR layers equal to two, four and eight, respectively. A scattering dip for case 1 is observed at 1.95 GHz, and it shows comparable scattering characteristics to the uncloaked metallic target. Case 2 reduces the SCS of the target significantly, and a well-defined resonant scattering reduction is observed. Case 2 shows a maximum scattering reduction at 1.9 GHz of − 6 dB. Due to the increase in the mutual coupling between the SRR elements, a significant redshift is observed for case 3. The resonance is observed to be at 1.6 GHz for this design, and since this design shows the maximum scattering reduction, it is taken as the optimum design (around − 10 dB) in the experimental setup.

At 1.6 GHz, a full-wave simulation using CST Microwave Studio is performed for three cases, namely the bare metallic case, the metallic object covered by a constant surface admittance as in Eq. (12) and the SRR cloak as reported in Fig. 3c. These structures are analyzed independently in the simulation by exciting the complete structure with a plane wave, with its electric polarization oriented according to a TM definition, coming from the left to the right of each drawing. To understand the cloaking operation, the electric and magnetic field distributions are studied for the cloaked and uncloaked targets. Figure 5 shows the simulated electric field (real part) on the uncloaked target (a and b), cloaked object covered at $b=13$ mm (the radius of the circle passing from the centers of the SRRs) with a surface impedance equal to $Z_s=- j148.8 \Omega$, according to Eq. (12) (c and d) and the 8-layer cloak with SRRs (e and f). One could observe from Fig. 5a,b that the uncloaked cylinder blocks electromagnetic power flow and a shadow is observed behind the target.

The computed field distributions for the cloaked target (case 3) are shown in Fig. 5c–f for a surface admittance cloak and the SRR cloak. A flow of electric fields is observed in the computational domain, as shown in Fig. 5(middle) and (bottom) respectively. This means that the cloaking structure significantly reduces scattering from the metallic target under consideration and the shadow observed in the uncloaked target, breaking the field lines, is absent in the cloaked scenario since both surface admittance and SRR cloak bends the incident electromagnetic wave into the output face, and no scattering is observed around the cloaking layer. Similar results are observed for the magnetic fields in Fig. 5b,d,f where there is a clear correspondence between the scattering from a homogeneous surface impedance and its realization with SRRs. Field lines are restored behind the structure and high magnetic discontinuity is observed in both covered cases.

**Figure 6:**
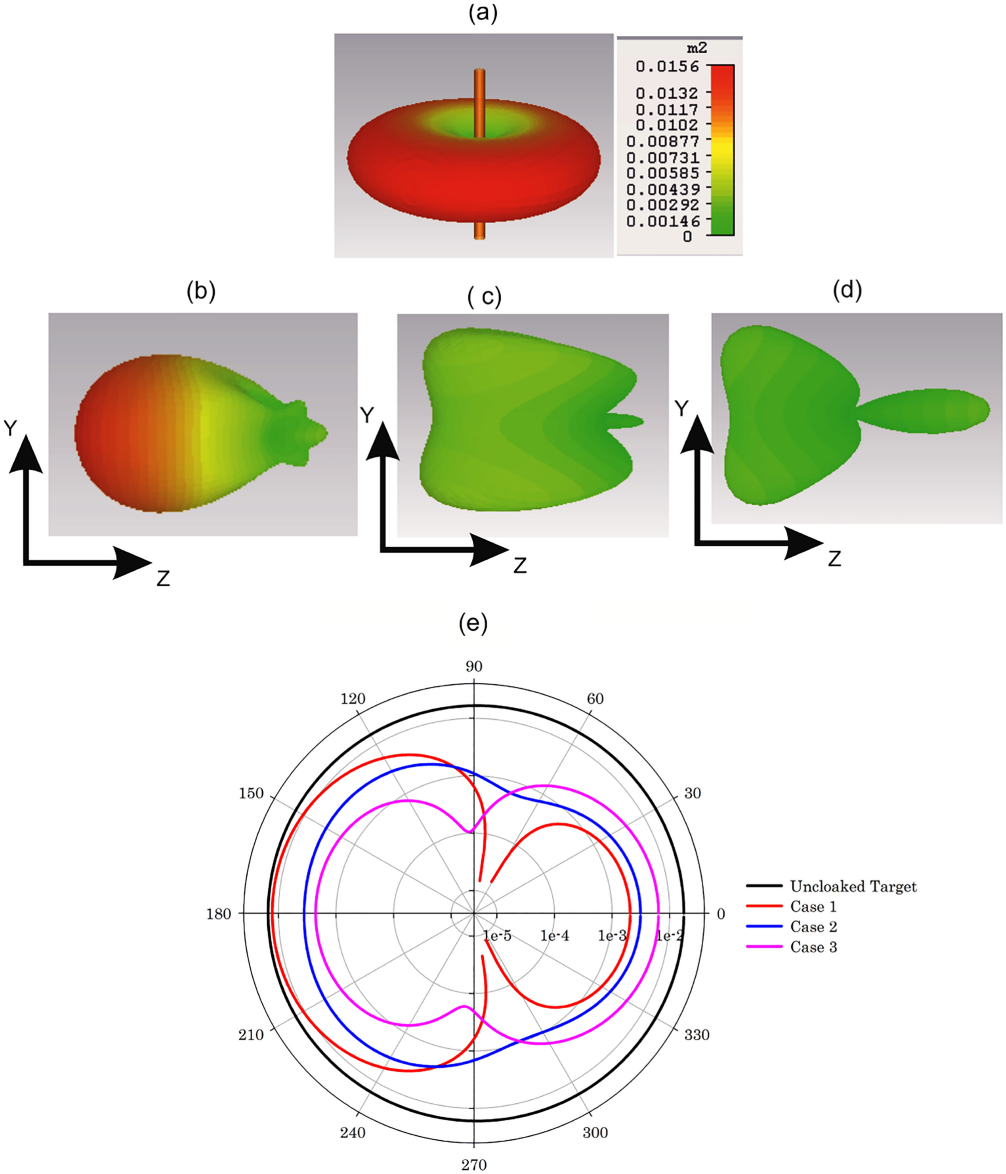
Scattering patterns of the cloaked and uncloaked targets (**a**) uncloaked target and (**b**) cloaked target 2-layers (Case 1), (**c**) cloaked target 4-layers(Case 2), (**d**) cloaked target 8-layers (Case 3) and (**e**) normalized scattered power along the azimuth plane of the three cases.

The scattering characteristics of the cloaked and uncloaked targets are also verified by computing the 3D scattering patterns. Fig. 6a shows the scattering pattern of the uncloaked target. The uncloaked target scatters electromagnetic power equally in the azimuth plane and two principal nulls are observed along the elevation plane. The non-resonant electric dipole excitation is responsible for this scattering mechanism, and hence the target is detectable from far-field scattering measurements. Figure 6b–d shows the scattering pattern of the three cloaked targets. One could observe from Fig. 6d that the eight-layer design (case 3) significantly reduces scattering in comparison with the other two cases. Please note that we have used the same scale for all four cases. Figure 6e illustrates the scattered power along the azimuth plane of the three cases normalized with that of a the bare metallic cylinder. Case 3 shows a minimum scattering compared to the other two cases under study.

### Experimental prototype

For experimental and simulation studies, we constructed three prototypes. The first one, designated as case 1, consists of a cylindrical metallic target surrounded by only two SRR strips, as sketched in Fig. 3a. The azimuthal displacement between the two strips is $180^\circ$. A column of $8 \times 1$ of SRR cells is arranged vertically in a strip, and the assembly consists of 16 SRR inclusions.

**Figure 7:**
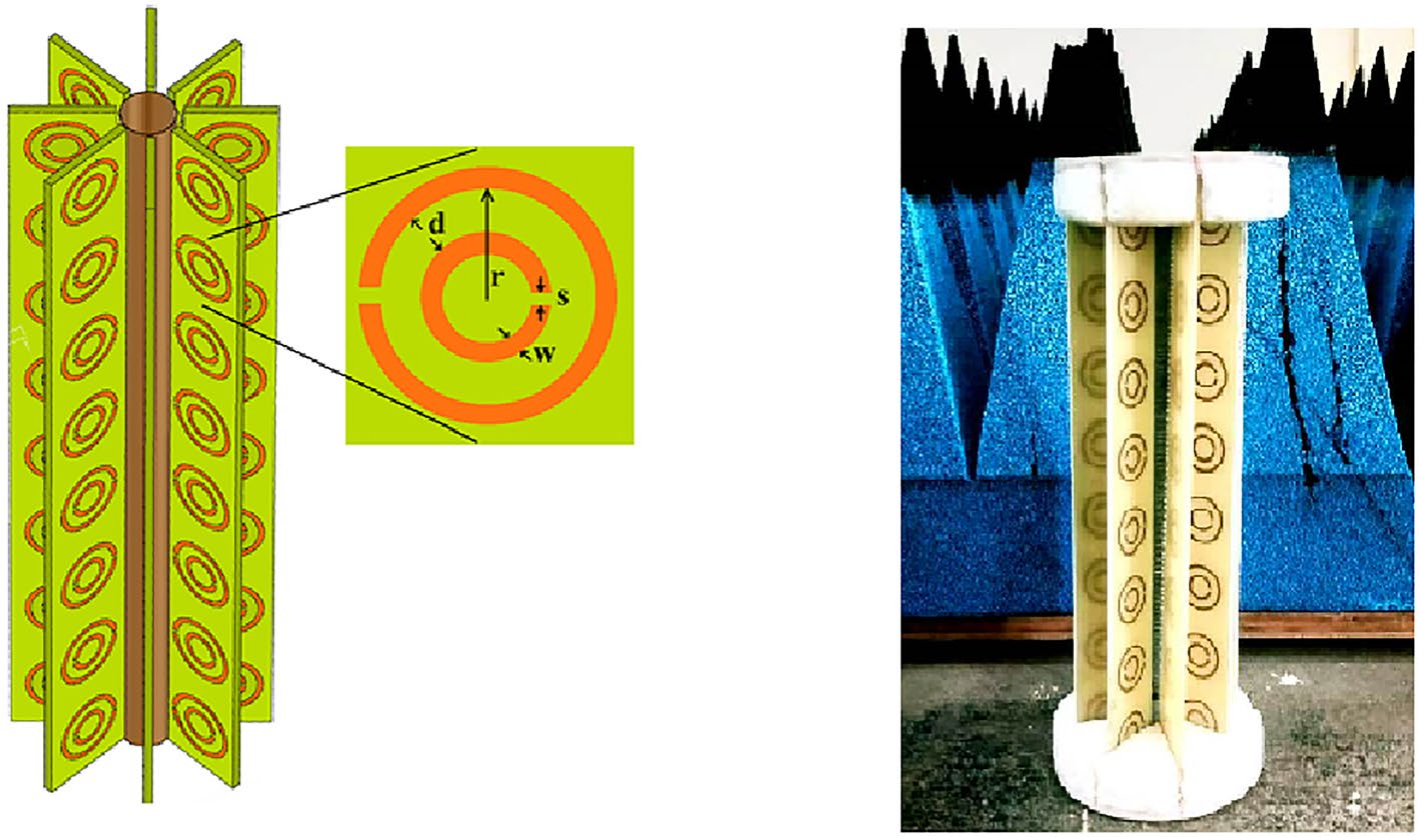
Sketch (left) and experimental sample (right) of the 8-layer SRR cloak.

The length of the target cylinder is 160 mm, and its radius is $a=5$ mm. The dimensions of the SRR used, printed on substrate using PCB technology and vertical periodicity $p=20$ mm, are shown in the inset of Fig. 7 and in particular, they are (unit of measure is millimeter) shown in Table 1.

**Table 1:** Parameters of the SRR.

r	d	s	w
6.7	2	0.8	1

**Figure 8:**
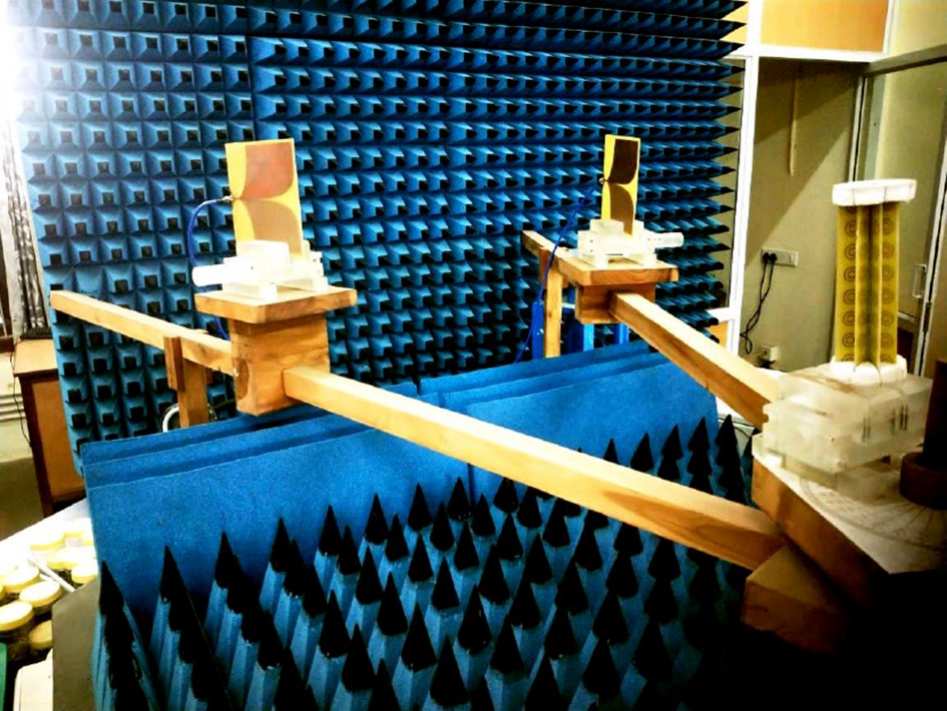
Backscattered measurement setup.

In the second design (case 2), we used 4 SRR strips arranged around the target, as shown in Fig. 3b. The total number of SRRs in this design is 32. In the third design (case 3), the number of SRR strips is increased to 8, as shown in Fig. 3c and detailed in the experimental sample in Fig. 7. The SRR strips are printed on an epoxy substrate of dielectric constant 4.4 and height 1.6 mm using the standard photo-lithographic procedures. The thickness of the implanted Copper metallization is 35 $\upmu$m. The scattering characteristics of the three prototypes are verified using backscattering measurements using a network analyzer. To measure backscattering, the uncloaked target is placed at the center of a turntable assembly as reported in Fig. 8.

Two ultra-wideband antennas are utilized for the measurement. One antenna is configured in the transmission mode and the second one is in the reception mode. A THRU calibration is performed by connecting the two RF cables, and the Frequency Gated by Time (FGT) calibration is applied to notch-out unwanted noise signals received from other directions. The resultant received power is taken as the reference, and finally, the cloaked target is placed instead of the bare metallic target.

**Figure 9:**
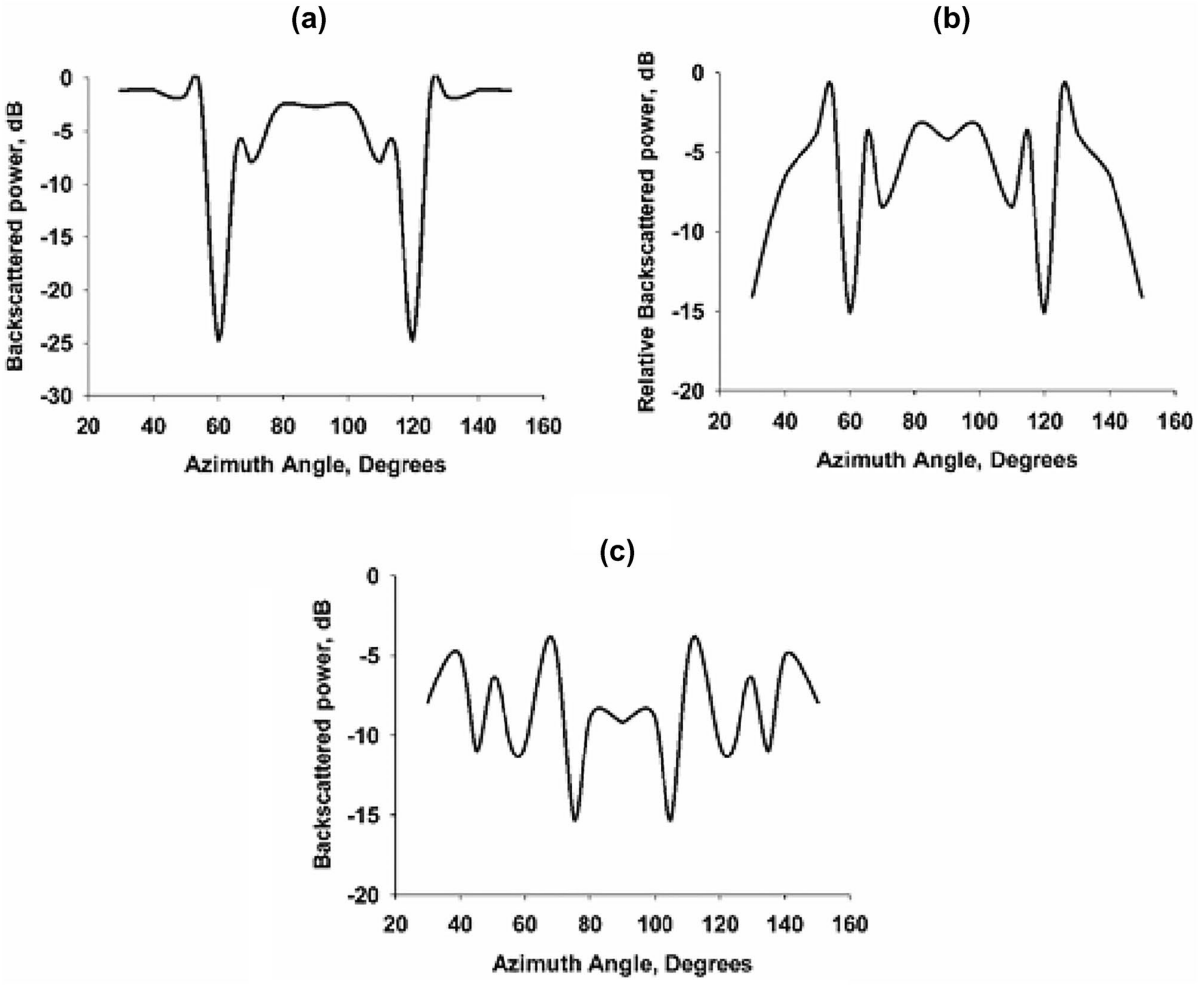
Back-scattered power from the fabricated samples (**a**) Case 1 (1.95 GHz), (**b**) case 2 (1.9 GHz), and (**c**) Case 3 (1.6 GHz).

The backscattered power thus received is recorded using the interface computer. For bistatic measurements, the receiving horn antenna is rotated along the azimuth angle using the turntable assembly, and the corresponding backscattered powers are recorded for the three fabricated prototypes. Since the eight-layer structure (case 3) shows a better reduction in backscattered power, it is taken as the optimum design. Figure 9 shows the backscattered power from the fabricated designs. Figure 9a shows the backscattered power from the two-layer (Case 1) design. It is evident that the design shows poor backscattering characteristics compared to the other two designs. For this case, the backscattered power is comparable in magnitude for most of the azimuth angle as that of the bare metallic target. It is interesting to note that as the number of SRR layers around the target increases, the backscattered power is decreased, as shown in Fig. 9b,c. This can be interpreted as a better equivalent surface admittance at the boundary $r=b$ passing from the centers of the SRR. Case 2 shows better backscattering characteristics compared to case 1. A significant reduction in backscattering is observed for the eight-layer design (Case 3) compared to the other two designs. A maximum backscattering reduction of the order of − 16 dB is observed for this design.

**Figure 10:**
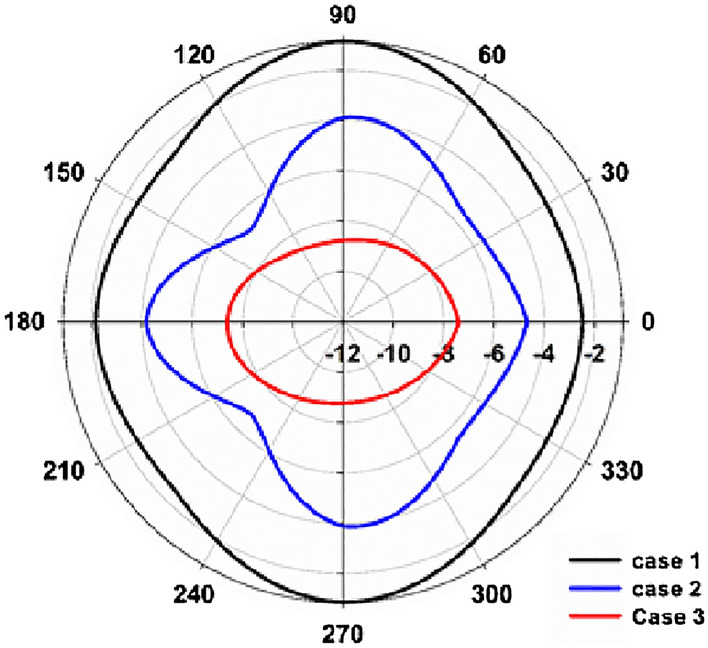
Monostatic scattering characteristics of the fabricated configurations at the scattering dips.

We have also performed the Monostatic scattering measurements to compare the scattering performance of the three fabricated prototypes. In monostatic measurements, the power received from the uncloaked target located at the turntable assembly center is taken as the reference after performing the FGT calibration. The cloaked target is rotated along the azimuth plane while two UWB antennas are kept fixed. The received power thus obtained is plotted in Fig. 10 for the three configurations under study. It is evident from the graph that the eight-layer configuration (case 3) gives a better backscattering reduction for all azimuth rotations of the target. It is also noted that the two-layer (case 1) and four-layer (case 2) structures also exhibit scattering reduction compared to a bare metallic target.

## Discussions with multipole theory and parametric analysis

To clarify the reason behind this peculiar scattering behavior, multipole scattering theory has been utilized to retrieve the structure’s resonant mechanism^23^. The multipolar decomposition provides an in-depth description of the scattering properties of the composite due to the induced charge-current distributions. Scattered power from the induced multipoles could be calculated by integrating spatially distributed current distributions of the unit cell. The principal multipole amplitudes—involved here can be calculated as

14 $$\begin{aligned}&\vec {P}=\dfrac{1}{j\omega } \int {\vec {J}d^3r}, \end{aligned}$$

15 $$\begin{aligned}&\vec {M}=\dfrac{1}{2c} \int {(\vec {r} \times \vec {J})d^3r}, \end{aligned}$$

16 $$\begin{aligned}&\vec {T}=\dfrac{1}{10c} \int {\begin{bmatrix} (\vec {r} \cdot \vec {J}) -2r^2\vec {J} \end{bmatrix} d^3r}, \end{aligned}$$

where $\vec {P}$ is the electric dipole moment, $\vec {M}$ is the magnetic dipole moment, $\vec {T}$ is the toroidal moment, *c* is the speed of light in vacuum, *r* is the displacement vector from the origin and *J* is the current density retrieved from simulations. The total power radiated from different multipole moments can be formulated as

17 $$\begin{aligned} I=\dfrac{2\omega ^4}{3c^3}|P|^2+\dfrac{2\omega ^4}{3c^3}|M|^2+\dfrac{2\omega ^6}{3c^5}|T|^2+\cdots . \end{aligned}$$

**Figure 11:**
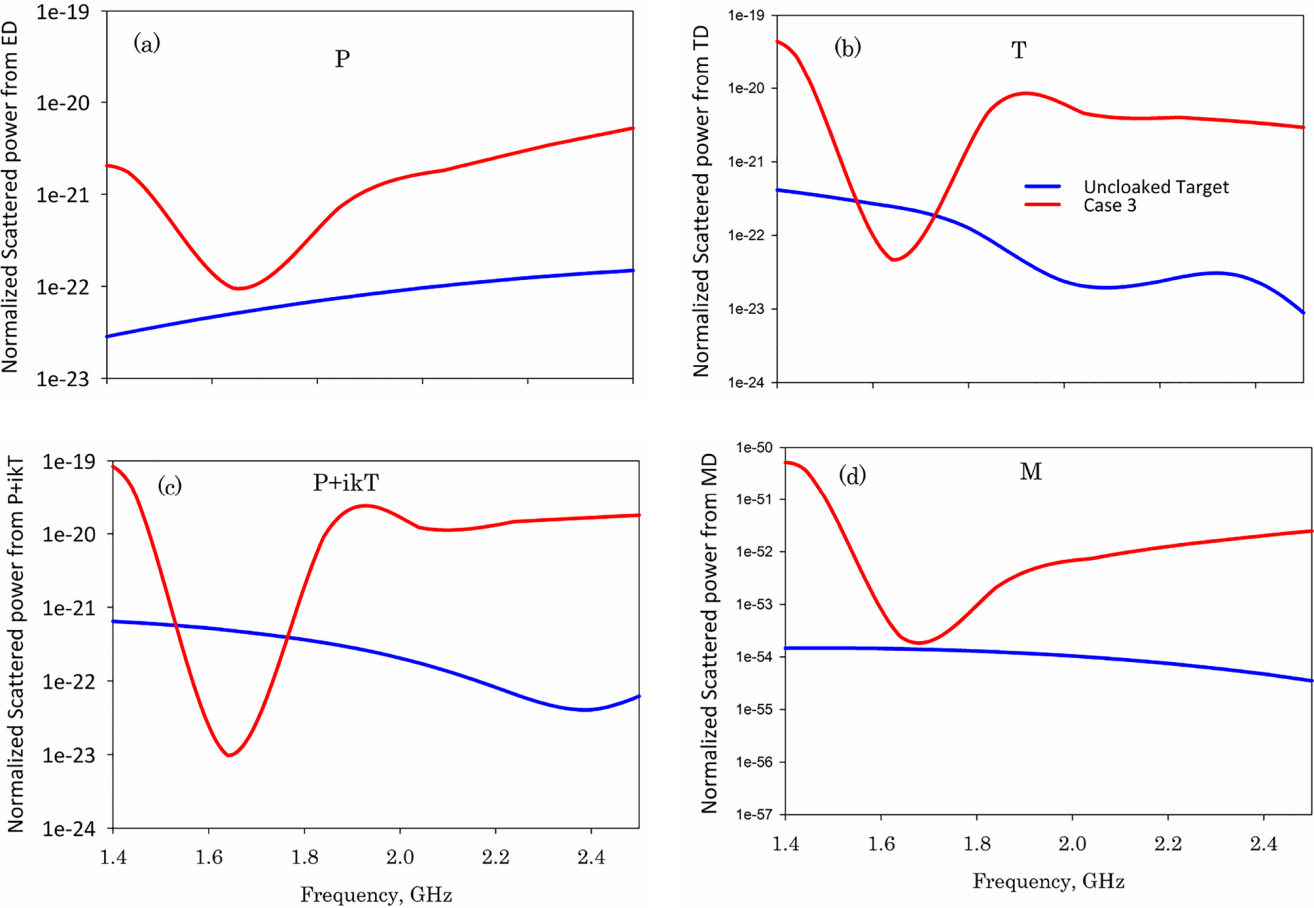
Normalized scattered power from various multipoles for the studied structures target only (blue curve) and Case 3 (red curve): (**a**) dipole moment P, (**b**) toroidal dipole T, (**c**) anapole state P+ikT and d) magnetic dipole moment M.

We have retrieved the scattered power contribution from the electric, magnetic, and toroidal moments for the uncloaked and cloaked cylinders and the results are shown in Fig. 11. The bare uncloaked metallic cylinder is characterized by the non-resonant contribution from the electric dipole moment. The entire spectrum is dominated by the scattering from the electric dipole moment and hence the scattering pattern looks like that of an electric dipole as shown in Fig. 6a. The scattering contribution from the magnetic dipole moment is insignificantly less and hence could be neglected. Adding SRR around the metallic cylinder excites resonance as indicated by the solid red lines in Fig. 11. In an SRR, both electric and magnetic resonances could be excited depending on the nature of the impinging plane wave. When the incident electric field is oriented along ${\hat{z}}$ and excites the slits, with the magnetic field perpendicular to the axis of the SRR, the magnetic moments can be excited on a circumference passing from the centers of the SRR and the toroidal dipole resonance is excited as in case of a cluster of four dielectric rods, and a metallic cylinder at the center^20^. The electric dipole resonance is created by the time- varying positive and negative charge distributions on the lower and upper SRR elements and the magnetic resonance is created by the circular current loops on the SRR elements. Due to the mirror symmetry arrangement of the SRR elements, the excited electric and dipole moments are excited out-of-phase. The out-of-phase oscillation of electric dipole moments excited on the SRR and the metallic target cancels the radiated power from the electric and toroidal dipole moment ($P+jk_0 T$) for the three cases. On the unit cell, in subwavelength condition, this corresponds to impressing a set of currents whose net sum is zero, obeying to Kirchhoff current law^29^ The excitation of the anapole condition is confirmed by the scattering dip observed for the electric and toroidal dipole moments around 1.6 GHz for case 3. This anapole condition reduces the scattering from the electric dipole moment significantly, resulting in electromagnetic invisibility. This technique is similar to the recently observed anapole-based cloaking system, in which scattering reduction is achieved by the destructive interference between the electric and toroidal dipole moments, as reported in the literature^20^ with dielectric rods to form the cloak.

**Figure 12:**
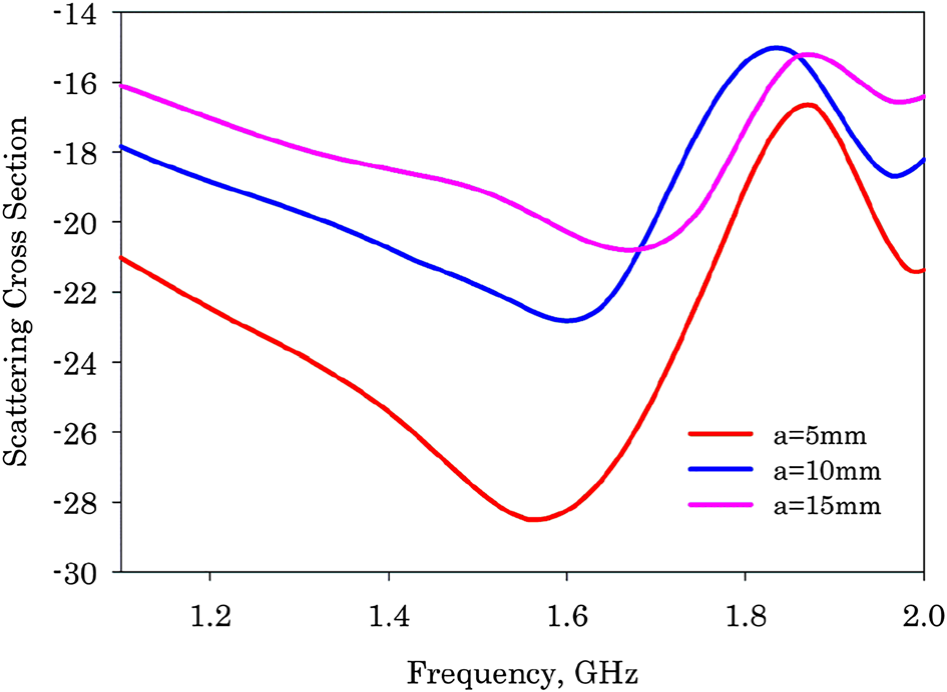
Monostatic scattering characteristics of the fabricated configurations at the scattering dips.

Parametric analysis has been performed to find out the effect of the radius of the metallic cylinder target on scattering behavior and these results are shown in Fig. 12. It is observed that as the radius of the target is increased, the resonance shows a blue shifts towards the higher side of the spectrum and scattering from the structure is found to be increased. This is due to the fact that an increase in the radius of the target excites a significant scattering from non-resonant electric dipole moments and the contribution from the resonant toroidal moment is found to be decreased thereby violating the condition for anapole excitation.

## Conclusions and future work

This paper has studied and presented the experimental verification of an anapole-based cylindrical electromagnetic cloaking scheme using a Split-Ring Resonator array in the microwave regime. The excitation of the anapole state significantly reduces the scattering from the electric and toroidal dipole moments contributions at the far-field resulting in a reduction in the Scattering Cross Section of the target, behaving like covered by a homogeneous surface impedance cloak in Mie theory. These results are verified using the multiple scattering theory and experimentally verified in the microwave regime inside an anechoic chamber. Future works will include a study on the geometrical parameters of the SRR as a function of an equivalent closed-form surface admittance. In addition, a surface cloak with more than one harmonic suppression can be modeled for future investigations.

## Data Availability

The datasets used and/or analysed during the current study available from the corresponding author on reasonable request.
